# Author’s Reply: Hepatitis E Virus Infection in Iranian Kidney-Transplant Patients

**DOI:** 10.5812/kowsar.1735143X.790

**Published:** 2011-11-30

**Authors:** Zakieh Rostamzadeh Khameneh, Nariman Sepehrvand

**Affiliations:** 1 Department of Microbiology, Urmia University of Medical Sciences, Urmia, Iran; 2 National Institute of Health Research, Tehran University of Medical Sciences, Tehran, IR Iran

**Keywords:** Kidney Transplantation, Hepatitis E, Iran

Dear Editor,

On behalf of all of the coauthors, I would like to thank Dr. Kamar for his interest in our study. In the letter, Dr. Kamar summarized the most important findings on hepatitis E infection in organ transplant recipients [1], most of whom have relied on the valuable contributions by of Dr. Kamar’s group in France [2][3][4]. In our study, we noted a high seroprevalence of anti-HEV IgG. Almost 30% of transplant recipients were seropositive for anti-HEV IgG [5]. We also found unexplained increases in liver function tests in transplant recipients. However, there was no significant difference in serum alanine transferase (ALT) levels between anti-HEV-seropositive and -seronegative groups [5].

As mentioned in the letter, serological methods have certain limitations. There are doubts regarding the diagnostic value of anti-HEV IgG serological evaluation in the diagnosis of HEV infection. In a study in Taiwan, an area in which hepatitis E is not endemic, the sensitivity of anti-HEV IgG compared with reverse-transcription PCR was 86.7% [6]. Its specificity in diagnosing acute hepatitis was 92%. Lin et al. concluded that anti-HEV IgG is a good test for screening acute hepatitis E in nonendemic areas [6]. Jiang et al. evaluated the quality of diagnostic ELISA kits in detecting HEV-specific IgG using HEV diagnostic reference sera from positive and negative cases, observing that the conformity of positive results exceeded 90% in all kits [7]. In contrast, Zaki et al., in Egypt, an endemic area for hepatitis E, found the sensitivity of anti-HEV IgG to be very low (2.3%) [8]. It appears that the diagnostic value of anti-HEV IgG serological tests in endemic areas is questionable.

As emphasized by Dr. Kamar, the setting of transplant recipients in Iran requires further evaluation using more specific modalities, such as polymerase chain reaction (PCR). Studying the presence of HEV RNA, its relationship with elevated liver enzymes, and acute or chronic forms of infection in these patients is recommended.
